# Supplementary material for: Are carbon emissions trading and green financial instruments synergistic? -Comprehensive quantitative research based on content analysis
Source: PLoS One. 2024 Mar 7;19(3):e0298601. doi: 10.1371/journal.pone.0298601 (PMC10919601; doi:10.1371/journal.pone.0298601)
Supplement: S1 Data — (ZIP) [file pone.0298601.s001.zip › Data/地级市Z_F工作报告.zip!/║╙─╧/╓ú╓▌╩╨/└┤╫╘╣1⁄2╓┌║┼í╛┬φ┐╦╩2╛▌═°í┐_2013.docx]

各位代表：

　　现在，我代表市人民政府，向大会作政府工作报告，请予审议，并请各位政协委员和列席人员提出意见。

　　一、2012年工作回顾

　　过去的一年，在省委、省政府和市委的正确领导下，全市上下按照“全国找坐标、中部求超越、河南挑大梁”的总体要求，认真落实《中原经济区郑州都市区建设纲要》，编制实施郑州都市区建设三年行动计划，科学谋划并大力推进“三大主体”工作，着力“保增长、调结构、促转型、惠民生”，较好地完成了市十三届人大四次会议确定的目标任务。

　　——经济平稳较快增长，质量效益持续提升。面对严峻的经济下行压力，不断加大经济调节力度，出台了一系列保增长的政策措施，全市经济社会发展继续保持了良好态势。初步统计，全市生产总值达到5547亿元，比上年增长12%；规模以上工业增加值完成2614亿元，增长17%；公共财政预算收入完成606亿元，增长21%；固定资产投资达到3561亿元，增长23%；社会消费品零售总额达到2290亿元，增长15%；居民消费价格指数涨幅为2.7%；城镇居民人均可支配收入24246元，增长12.2%，农民人均纯收入12531元，增长13.4%。

　　——新型城镇化强力推进，城乡一体化进程持续加快。以六个切入点为载体，加快推进新型城镇化建设，实现了全域拆迁面积、城乡基本建设投资、土地收储和争取土地指标三个突破性进展。“两环十七放射”开工建设总量达70%以上，十条市域快速通道中郑汴物流通道、南三环东延、四港联动大道、国道107线、郑新快速通道新郑段建成通车。国家、省、市三级交通项目完成投资263亿元，是2011年的1.5倍。生态廊道绿化面积完成3600万平方米。中心城区功能逐步提升，“六旧九新”片区改造启动项目302个，三环以内177家市场完成外迁23家，启动外迁25家。四类社区建设全面展开，启动城中村改造项目60个、合村并城社区98个、新型农村社区68个、新市镇25个，建成安置房800多万平方米。畅通郑州建设扎实推进，地铁1号线顺利实现双线轨通，京广高铁全线通车，郑州东站建成投用，京广快速路一期建成通车，打通断头路20条，新建停车场367个，新增停车泊位7万多个。三环快速化工程、高速路出入市口、南水北调跨渠桥梁等重大基础设施工程进展顺利。城区道路大修、排水管网改造、污水、热力、电力等市政工程有序推进，南部热源厂正式运行，城市管理整治提升初见成效。郑东新区实现“十年建新城”目标，郑州城市形象不断提升。城市组团加快建设。积极创建国家森林城市，完成造林22万亩，新增城市绿地1067万平方米，文博森林公园、西流湖生态公园（北区）建成开园。生态水系和水源工程进展加快，西流湖污水治理初见成效，龙湖调蓄工程建成蓄水。PM2.5监测启动运行。

　　——现代产业体系加快构建，结构调整效应持续显现。积极破解影响郑州长远发展的深层次结构性矛盾问题，明确了工业和服务业主导产业定位及布局，投资2268亿元、开工建设了211个省市重点项目，产业转型升级步伐进一步加快。新型工业化主导地位增强。七大工业主导产业对工业增长贡献率达到83%，拉动全市工业增长14个百分点。传统产业改造提升成效显著，战略性新兴产业和高新技术产业快速发展。汽车及装备制造、电子信息等八大产业基地加快整合。国机集团、华强电子、海尔产业园、中国联通中原数据基地等一批项目开工建设，格力电器等重大工业项目建成投产。销售收入超百亿元企业达到11家，郑煤机集团、华北石油局超100亿元，宇通集团首次突破300亿元，鸿富锦精密电子（郑州）有限公司突破1000亿元。产业集聚区工业企业主营业务收入4500亿元，增长26%，航空港产业集聚区成为全省第一个千亿级产业园区。现代服务业发展逐步提速。全年服务业投资实现2127亿元，增长26%。郑东新区金融集聚核心功能区扎实开局，龙湖金融中心首批签约16个金融项目。商贸服务业和文化旅游业提档升级，房地产业健康发展。华南城、金马凯旋家居CBD等项目开工建设，方特欢乐世界、二七万达广场、王府井百货等项目建成运营，中石化郑州分公司、丹尼斯百货销售收入首次突破100亿元。都市型现代农业稳步发展。粮食总产量达到170万吨，实现“十连增”。“菜篮子”产品产量稳步提高。现代农业示范区“136”工程全面启动。改造中低产田20万亩，新增有效灌溉面积10万亩，改善节水灌溉面积7万亩，农业生产条件持续改善。三全、好想你等五大农业产业化集群初具规模，中牟·国家农业公园形象初显。全市农产品加工型龙头企业实现销售收入470亿元，5家被列为全国第一批主食加工业示范企业，占总数的四分之一。

　　——开放创新双驱动战略深入实施，城市核心竞争力持续增强。成功举办了第18届郑交会、壬辰年黄帝故里拜祖大典、第九届中国郑州国际少林武术节、第四届中国·郑州农业博览会等一系列重大活动，全年会展面积达到175万平方米，郑州的影响力进一步提升。全年引进市外境内资金1099亿元人民币，约占全省总量的四分之一；实现外商直接投资34.3亿美元，约占全省总量的三分之一。产业招商成效明显，新签约亿元以上项目145个。出口总值完成202亿美元，增长1倍以上。全国跨境贸易电子商务服务试点项目启动。全市研发投入占GDP比重达到1.7%，远高于全省平均水平。高新技术产业产值增长32%，专利申请量增长47.5%，专利授权量增长47.6%。国家技术发明奖实现了零的突破。国家专利审查协作河南中心落户郑州。荣获国家创新型试点城市、国家知识产权示范城市称号。

　　——社会事业全面发展，人民生活持续改善。坚持经济发展与民生改善相统一，全市财政用于改善民生的支出达512亿元，增长23%，占公共财政预算支出的73%。民生“十大实事”基本完成。新增城镇就业再就业13万人，转移农村劳动力11万人。新建、改建农村公路500公里。解决49万农村人口饮水安全问题。新建、改扩建中小学校40所、幼儿园96所，新增市区中小学优质学位5万个、幼儿园学位2.5万个。完成易地扶贫搬迁和整村推进年度任务，扶贫标准提高到3000元，比国家和省定的标准高700元。

　　医疗服务保障能力持续提升，一批优质医疗资源项目投入使用，“片医负责制”社区卫生服务已覆盖全市800多万城乡居民，全国首家启动实施“居民健康卡”，新农合经办机制改革“郑州模式”在全国推广。社会保障水平不断提高，基本实现了应保尽保。社会救助工作进一步加强。保障房建设完成省定的目标。入选国家首批“公交都市”建设示范工程创建城市。国家公共文化服务体系示范区创建工作深入推进，文化惠民工程顺利完成。深入开展“社区管理年”活动，社区建设管理水平不断提升。颁布了郑州市妇女儿童十年发展规划，妇女儿童事业全面发展。低生育水平持续稳定，我市荣获“全国人口和计划生育工作先进集体”。

　　——政府职能进一步转变，民主法制和社会管理持续加强。自觉接受人大及其常委会的监督，认真执行人大及其常委会的决议决定；主动接受人民政协的民主监督，认真听取民主党派、工商联、无党派人士和各人民团体的意见和建议。全年办理人大代表议案7件、建议543件，满意率接近100%；办理政协委员提案468件，办结率100%，满意率达到了98.5%。坚持依法行政，建设法治政府，推进依法治市，制定政府规章6部，提请市人大常委会审议地方性法规草案3部。积极建立以网格为载体的坚持依靠群众推进工作落实长效机制，政府职能在基层得到进一步强化。下沉各级网格工作人员4.5万人，围绕7个重点领域和城市管理整治提升13项工作，及时排查化解了一大批热点难点问题，政府职能部门和公职人员履职意识、责任意识有了明显提高。加强和创新社会管理，深化社会治安综合治理，狠抓安全生产、消防安全和食品药品安全监管，深入推进平安郑州建设，社会大局和谐稳定。

　　加强社会主义核心价值观教育和精神文明创建，公民道德和社会文明程度持续提高。国防教育和国防后备力量建设取得新的成效，创建全国双拥模范城实现“六连冠”。外事、侨务、对台、民族、宗教、统计、审计、档案、史志、气象、地震、人防、社科等工作都取得了新的成绩。对口支援新疆哈密地区的工作成效明显。

　　回顾过去一年的工作，我市在发展的各个领域都在过去的基础上形成了比较系统的思路，郑州发展的基础更加稳固、发展的机制更加完善、发展的空间更加广阔，郑州在中原经济区建设中的战略地位越来越突出，科学发展的前景越来越美好！

　　各位代表！

　　在复杂严峻的形势下取得这些成绩，确属不易。这是省委、省政府和市委正确领导的结果，是人大、政协和社会各界大力支持的结果，更是全市人民群策群力、团结拼搏的结果。在此，我代表市人民政府，向辛勤工作在各行各业的广大干部群众，向驻郑部队、武警官兵、公安民警以及社会各界人士，向关心支持郑州发展的港澳台同胞、海外侨胞和朋友们，表示崇高的敬意和衷心的感谢!

　　当前，郑州的发展已站在了新的历史起点上，但我们也清醒地看到经济社会发展中存在的困难和挑战。主要是：经济发展的结构性矛盾依然突出，经济增长的质量和效益不高；农业基础还相对薄弱，农村发展相对滞后，农民持续增收的难度加大；基础设施和公共服务跟不上城市发展的速度，交通拥堵、环境污染等一些群众普遍关心关注的问题还需要下大工夫解决，就学、就业、就医等一些涉及群众切身利益的问题还需要进一步做实做好；一些政府工作人员的履职能力、勤政廉政意识和依法行政水平还需要进一步提升等。对此，我们将高度重视，不断认真地加以解决。

　　二、2013年工作总体要求和主要目标

　　2013年，郑州发展仍处于重要的战略机遇期。国内外产业向中西部加速转移，中部地区已经进入开放招商、承接产业转移的“黄金期”。郑州区位交通、人力资源、消费市场、基础支撑条件等承接产业转移的比较优势突出。同时，重要战略机遇期的内涵和条件发生了深刻变化。我们面临的机遇不再是传统机遇，而是倒逼我们扩大内需、提高创新能力、促进发展方式转变、提高增长质量和效益的新机遇。我们必须顺势而为，乘势而上，努力在错综复杂的经济环境中实现更好更快的发展。

　　做好今年的政府工作，我们要深入学习贯彻党的十八大精神，围绕主题主线，以提高经济增长质量和效益为中心，以“强投资、夯基础、调结构、求提升，努力实现经济社会持续健康快速发展”为总要求，强化三大主体工作，推行三大机制，抢抓机遇，持续求进，加快推进中原经济区郑州都市区建设三年行动计划，全力建设郑州航空港经济综合实验区，着力保障和改善民生，为率先全面建成小康社会奠定坚实基础。

　　今年经济社会发展的预期目标是：地区生产总值增长12%以上，地方公共财政预算收入增长13%以上，固定资产投资增长25%以上，规模以上工业增加值增长17%以上，社会消费品零售总额增长16%左右，出口总额增长25%左右，外商直接投资增长11%左右，城镇居民人均可支配收入增长12%左右、农民人均纯收入增长12%左右，人口自然增长率控制在6‰以内，节能减排完成省下达目标。

　　实现上述要求和目标，我们要按照市委部署，坚持“总要求”，站位“全国找坐标、中部求超越、河南挑大梁”，抢抓《中原经济区规划》获批、郑州航空港经济综合实验区上升为国家战略的重大历史性机遇，不断强化郑州在全省的重要、重点、重心地位，努力在中原经济区建设中“挑大梁、走前头、作表率”。强化“三大主体”工作，强力推进新型城镇化引领、现代产业体系构建和以网格为载体坚持依靠群众推进工作落实长效机制“三大主体”工作，努力在加快信息化进程中率先走出一条“两不三新”三化协调科学发展之路。推行“三大机制”创新，大力推行城乡规划土地管理城市建设“六统一”工作机制、推行产业集聚区与行政区套合机制和 “重基层、重实干、重实绩”的人才培养使用管理机制，积极破解土地、资金、人才等要素制约。突出“四个着力”，着力强投资，着力夯基础，着力调结构，着力求提升，不断增强郑州在中原经济区建设中的龙头作用、重心作用和示范带动作用！

　　三、2013年工作重点

　　今年，我们将重点做好以下工作：

　　（一）深入推进新型城镇化建设，为经济社会持续健康快速发展提供广阔空间。坚持新型城镇化引领郑州都市区建设，按照“一主三区四组团、36个重点产业集聚区、27个新市镇、183个新型农村社区、56个历史文化风貌特色村”的空间布局，加快城镇化进程，提升城镇化质量。加快重点区域开发建设。大力实施“东扩西拓南延”，不断拉大城市框架。加快郑东新区建设，重点抓好龙湖工程、龙湖CBD、龙子湖湖心岛、郑州东站等重点区域基础设施建设。做好省级和市级公共文化服务区规划，启动一批基础设施和公共服务设施项目。进一步完善经开区、高新区基础设施配套服务功能。加快城市周边新区建设，实施一批城市组团建设项目，不断提升城市功能和形象。深入推进畅通郑州建设。确保年底前轨道交通1号线投入运营，2号线一期主体结构完工，开工建设1号线二期和5号线，力争轨道交通二期工程获批。加快建设“井字+环线”快速路网系统，确保年底前三环快速化工程主体完工，开工建设京广快速路二期工程、陇海快速路、金水路、花园路、紫荆山路准快速化工程。完成文化路与连霍高速、航海东路与京港澳高速等5座互通式立交出入市口建设项目，争取郑登快速路、陇海路西延、科学大道西延等市域快速通道部分路段通车，开工建设中原路西延至巩义段、大学路南延至省道S323段。加快南水北调41座跨渠桥梁主体工程建设。打通主城区断头路20条以上。新增停车泊位5万个。新建、改建县域路网500公里。改造提升中心城区功能。加快主城区15个片区和“一河两岸”整街坊整片区有机更新改造，以三环内批发市场、工业仓储、行政事业单位外迁为抓手，启动“六旧”片区改造项目22个、“九新”片区项目160个。完成50家市场外迁，启动30家市场外迁。加快商业综合体、地铁站周边、新建小区等商业网点规划建设。加大水、电、气、暖管网升级改造力度，加强市区道路、公交场站等市政设施建设和维护，持续开展城市管理整治提升活动，着重解决交通拥堵、占道经营、乱停乱放等突出问题。巩固国家卫生城市创建成果，抓好国家智慧城市试点工作。统筹推进城乡一体化发展。完善市县融合发展机制，壮大县域经济实力，着力培育和打造一批中小城市。加快六县（市）和上街区十字景观大道、中央商务区建设，抓好薛店镇等一批市级“三化”协调发展示范区建设，实施新市镇各类建设项目114个。进一步加快四类社区建设，积极推进城区、镇区、产业集聚区规划区周边合村并城和撤村并点工作，扎实做好群众拆迁安置工作，逐步实现县域村镇社区化。启动城中村改造项目31个、合村并城项目38个、新型农村社区项目24个。

　　（二）加快构建现代产业体系，为经济社会持续健康快速发展提供有力支撑。坚持以新型工业化为主导、服务业优先、都市型现代农业为基础，加快构建“特色鲜明、主业突出、布局合理、竞争力强”的现代产业体系。加快工业转型升级。壮大工业七大主导产业规模，加快建设八大千亿级产业基地。大力发展汽车及装备制造、电子信息等战略支撑产业，加快发展新材料、生物医药等战略性新兴产业，改造提升铝、食品、家居、品牌服装等传统优势产业。开展工业项目建设年活动，实施重点行业行动计划，推动一批重大项目建设。积极发展物联网、云计算、移动互联网、大数据等新一代信息技术，加快国家级“两化”融合试验区建设。实施万户中小企业创业培育计划、千名企业高级管理人员培训计划，新增高成长型中小企业500家、“专精特色”中小企业1000家。力争全年工业项目投入达到1800亿元、规模以上工业增加值突破3000亿元、七大主导产业实现销售收入突破9000亿元。促进现代服务业提速发展。围绕“一枢纽十中心”建设目标，大力实施“3551”工程。加快建设华南城、格力电器仓储等100个服务业重大项目，积极谋划中部无水港、机场快件中心、汽车服务业博览园、旅游集散中心等十大服务业项目。发展现代物流业，筹备建设中原物流网，争取年内开通直通欧洲铁路班列、澳洲全货机货运航线。加快郑东新区金融集聚核心功能区建设，开工建设一批金融项目。加快国家级商贸中心建设，力争将二七商圈打造成为千亿级商圈。加快中心商务功能区、特色商业区建设，力争6月底前规划通过省级审批。积极调整房地产市场供应结构，促进房地产业健康发展。加快发展文化创意旅游业，推动旅游产业转型升级。繁荣发展会展业，打造品牌会展和中部会展之都。深入推进服务业综合改革试点工作。力争全市服务业固定资产投资达到2600亿元以上，服务业增加值突破2500亿元，增速达到10%以上。强力推进重大项目建设。突出抓好国家、省、市重点项目建设，力争今年固定资产投资完成4500亿元。支持哈密至郑州800千伏特高压输电工程、郑徐高铁工程、南水北调郑州段，郑焦、郑开、机场城际铁路等重点项目加快推进。抓好旭飞光电“461”工程、中国联通中原数据基地、中国郑州国际汽车后市场产业园、中国龙工中原机械生产基地等投资超50亿元重大产业项目建设。加快产业集聚区建设。按照“四集一转”要求，加强重大项目调控和引导，促进同类项目向特色产业集聚区集中布局。加快建设综保区（航空港区）富士康IT产业园、国家生物医药产业基地，高新区IT产业园、经开区汽车及装备制造、现代物流等千亿级产业集群，提升配套服务功能。加快县（市、区）产业集聚区基础设施建设，以优良环境和配套服务吸引更多项目入驻。

　　（三）大力发展都市型现代农业，为经济社会持续健康快速发展提供坚实基础。始终把“三农”问题作为重中之重，认真落实强农惠农富农政策，持续加大投入，着力构建新型农业经营体系，强化都市型现代农业基础支撑，激发农村发展活力。以都市型现代农业示范区为载体，提升农业综合效益。加快实施都市型现代农业示范区“136”工程、高标准粮田万亩示范方工程，启动2～3个现代农业综合示范区、5个主导产业集聚区、10个特色产业园项目建设。支持农业产业化龙头企业加快发展，争创省级农业产业化集群，力争市级龙头企业达到410家，加工型龙头企业销售收入突破500亿元。继续扶持发展设施农业、休闲观光农业，切实抓好 “菜篮子”工程，开发新菜田4万亩，新增设施农业8000亩。以加强新型农村社区建设为切入点，改善农民生产生活条件。统筹各级财政支农资金支持新型农村社区建设，加快推进基础设施、公共服务向农村延伸。新增农村自来水用户6.5万户，建设小型水源工程5000个，解决27万农村人口饮水安全问题。改造中低产田20万亩，实现新增有效灌溉面积3万亩。启动农田水利现代化示范乡（镇）建设。开展农村环境连片整治和生态乡村创建活动，不断改善农村村容村貌。加大扶贫开发力度，全年完成5633户2万多人易地扶贫搬迁、20个贫困村整村推进的任务，确保贫困地区农民人均纯收入增长幅度高于全市平均增速。以体制机制创新为途径，推进农业经营方式转变。着力培育新型农业经营主体，推进标准化种养殖规模生产，加快构建集约化、专业化、组织化、社会化相结合的新型农业经营体系。大力推进主食产业化进程，促进粮油加工和粮食流通产业快速发展。加快40个农业技术推广服务区域中心站建设。实施以新型职业农民为重点的农村劳动力培训阳光工程，全年完成培训2万人以上。加强农资监管。完善农产品质量安全监管及可追溯体系，扩大农产品质量安全市场准入范围，将群众关注的原（杂）粮、干菜、干果、茶叶纳入市场准入检测监管。

　　（四）全力建设郑州航空港经济综合实验区，为经济社会持续健康快速发展提供新的有效载体。航空港经济综合实验区是我市扩大开放的重要平台，也是我市实现赶超跨越发展的优势所在。高标准编制实验区规划。围绕“建设大枢纽、培育大产业、塑造大都市”的战略定位，加快总体发展规划编制和报批工作，力争早日获批。加强实验区规划与郑东新区、经开区、新郑市、中牟县等区域相关规划的衔接，统筹重大产业项目布局和基础设施建设，推动区域一体化发展。扎实推进基础设施和公共设施建设。加快推进郑州机场二期工程、机场高速扩建、上街机场改扩建、双湖大道东延、新老107连接线、S102东段工程、四港联动大道南延等重点项目，打通综保区与主城区“四纵四横”联络通道，完善实验区陆路交通集疏网络。启动航空货站和实验区3平方公里核心区建设，推动起步区和重要功能区尽快成规模、出形象。着力引进高端产业项目。大力发展航空偏好型产业，重点承接航空产业、电子信息、生物医药等高端产业项目转移，加快发展通用航空产业，尽快形成以航空经济为主导的现代产业业态和产业体系。优先发展航空货运，继续推进与俄罗斯空桥、UPS、国货航等货运航空公司合作，力争新开货运航线7条。深化与富士康的合作，加快苹果手机制造、维修中心、电子部件全球采购、国际分拨中心建设。坚持“区港联动、区区联动”，提升新郑综合保税区辐射带动效应。力争全年实现生产总值230亿元，进出口总额300亿美元，机场旅客吞吐量达到1300万人次。

　　（五）进一步深化改革开放创新，为经济社会持续健康快速发展提供强大动力。改革、开放、创新是经济社会持续健康快速发展的强大动力，也是实现经济转型发展、赶超跨越的根本所在。推行“三大机制”创新。推行城乡规划土地管理城市建设“六统一”工作机制。按照“盘活资源、良性运行、政府主导、有序发展”的目标，建立“规划统一管理、土地统一收储、用地统一转让、补偿统一标准、收益统一分配、工程统一建设”的工作机制，优化资源配置，增强调控能力，调动市区两级加快发展的积极性。推行产业集聚区与行政区套合机制。按照“统一、精简、高效”的原则，充分发挥航空港经济综合实验区领导小组的作用，优化区域空间布局和资源配置，以实验区统筹郑东新区、经开区、综保区等区域协调发展，打造郑州改革开放的新高地；推进县（市、区）产业集聚区（专业园区）与所在乡（镇）街道办套合，规范管理体制，提高运行效能。推行“重基层、重实干、重实绩”的人才培养使用管理机制，注重在发展主战场培养使用人才，建立完善能力在一线培养、业绩在一线考核、人才在一线发现的人事管理制度。深化重点领域改革。积极稳妥推进事业单位分类改革和省直管县体制改革试点。深入推进财税管理体制改革，健全财力与事权相匹配的财政体制，提高科学理财水平。积极做好营业税改征增值税试点准备工作。扩大政府购买服务范围。积极推进金融创新，拓宽融资渠道，支持企业上市、发债融资，继续开展县（市、区）发行债券工作。统筹推进国有企业、医疗卫生、文化教育、收入分配和社会保障体制改革。加大产业招商力度。严格落实“五职招商”责任制和“三位一体”招商推进机制，瞄准国内外500强、大型央企和主导产业前20强企业，着力谋划和引进一批具有国际影响力、国内辐射力、国内外资源整合力的产业项目。力争全年开工100个5亿元以上、签约100个5亿元以上、谋划100个后续招商引资项目，实现招商引资总规模超过1600亿元。大力发展开放型经济。重点推进跨境贸易电子商务服务试点工作，培育国际网购物品集散分拨中心。加强电子商务监管，规范电子商务市场秩序。推动郑州铁路枢纽东联海港、西通中亚欧洲，加快建设国际货物集疏中心。拓展外需市场，扩大出口规模，积极承接出口加工型项目，加强汽车及零部件、铝精深加工、电子信息、纺织服装等出口基地建设。加快申建国家服务外包示范城市。力争全年进出口总额突破430亿美元。增强创新驱动力。以创新型城市建设为抓手，加快实施科技型企业成长路线图计划、研发中心建设等行动计划，促进科技型企业快速成长，新建产业技术联盟5个、市级研发中心300家，力争研发中心升格为省级50家、国家级3家。充分发挥驻郑高校、科研院所和企业的作用，加快推进未来科技城建设。深入实施知识产权战略，加快建设国家专利审查协作河南中心、国家知识产权创意试点园区。力争全市研发投入、专利申请量、专利授权量、科技成果量、技术合同成交额、高技术产业增加值实现新突破。科技进步对经济增长的贡献率达到58%。

　　（六）不断加强文化建设，为经济社会持续健康快速发展提供精神动力和智力支持。充分挖掘郑州深厚的历史文化资源，推动文化大发展大繁荣，努力打造华夏历史文明传承创新核心区。不断提升城市文明程度。巩固文明城市创建成果，扎实开展“道德讲堂”和志愿者活动，加强社会公德、职业道德、家庭美德、个人品德教育。完善社会信用体系，打造“信用郑州”。落实全民科学素质纲要。加快推进中原网二期工程建设，实施“郑州市百家网站建设推进工程”，开展“文明网站”创建活动。建立郑州市互联网站信息内容监管平台，不断净化网络环境。大力发展文化产业。扶持报业传媒、影视制作、动漫游戏、文化创意等文化产业发展。加快郑州华强文化科技产业基地、国家动漫产业发展基地、郑州信息创意产业园等产业集聚区建设。支持中原报业传媒集团、新华书店等国有控股企业发展壮大。扶持一批文化优势品牌做大做强。实施以“天地之中”为核心的城市整体营销策略，继续办好黄帝故里拜祖大典，展示郑州城市品牌。完善公共文化服务体系。创建全国公共文化服务体系示范区。规划建设市民公共文化服务区，加快推进现代传媒中心、文化艺术中心、文博展示中心、奥林匹克体育中心等项目的前期工作。大力实施文化惠民工程。实施文艺精品创作工程，推出一批有重大影响的文艺精品。加快大河村遗址博物馆二期工程建设，做好商城和大河村国家考古遗址公园项目的前期工作。编制五级健身休闲体系规划，完善城市体育设施，积极申办第九届城运会。

　　（七）努力打造美丽郑州，为经济社会持续健康快速发展提供生态安全保障。建设生态文明关系人民福祉、关乎郑州未来，我们要放在更加突出位置，不断增强可持续发展能力。加强资源能源节约。大力发展节能环保产业，实施一批节能重点工程，创建一批循环经济示范县（市）、示范园区和示范企业。加快“气化郑州”工程建设，落实差别化工业企业能源准入政策。稳步推进自来水、车用燃气等资源性产品价格改革。扎实推进新能源示范城市创建工作。加强环境保护。深化主要污染物排放总量预算管理，严格环境准入和污染物排放标准，适时开展排污权有偿使用和交易工作。实施生态环境综合治理，发布《“蓝天”工程白皮书》，加强PM2.5监测治理，加大汽车尾气、黄标车辆、工地扬尘、火力发电、燃煤锅炉污染的治理力度。严格环保执法，强化环境应急能力建设。加强水利保障。加强贾鲁河等重点流域综合治理，完成东风渠上游和潮河上游治理工程，治理水土流失面积80平方公里；实施南水北调配套工程、牛口峪引黄调蓄工程、邙山输水干渠复线工程，完善城市水源保障体系；完成龙湖蓄水、象湖开挖等生态水系建设工程；加快建设三环再生水管线、新力电力再生水利用等再生水利用项目，努力在水生态建设上实现新提升。加强生态建设。以创建国家森林城市和国家生态园林城市为载体，突出抓好生态廊道、森林公园、花卉苗木、园林绿化等重点工作，努力构建以道路景观绿化、河渠两岸绿化为骨架，以大面积生态林、公园、游园建设为重点，以庭院绿化、立体绿化、屋顶绿化、拆墙透绿为补充的都市区生态体系。加快推进郑州树木花卉博览园等15个森林公园、10个“走进森林”示范园及西流湖公园南区、南环公园、雕塑公园二期等大型综合公园建设。坚持城市绿地系统规划，严格城市园林资源管理，完善城市园林“绿色图章”制度。市区新增绿地500万平方米以上，营造生态林10万亩以上，建设花卉苗木基地2万亩以上。

　　绿城郑州、生态郑州、美丽郑州是我们共同的追求、美好的向往，也是所有郑州人奋斗的目标、共同的责任，让我们携手努力，共创宜居城市，同建美好家园！

　　（八）切实保障和改善民生，为经济社会持续健康快速发展提供和谐稳定的社会环境。强力推进民生工程。围绕与百姓生活息息相关的“柴米油盐酱醋茶、衣食住行教医保”，加大投入，办好省、市民生实事。千方百计扩大就业。实施创业富民计划，全年新增城镇就业再就业13万人，农村劳动力转移就业10万人以上，发放小额担保贷款16亿元。加大人力资源市场、零工市场建设力度，不断提高为外来务工人员的服务管理水平。切实增加居民收入。完善和落实工资决定机制、正常增长机制和支付保障机制，多渠道增加城乡居民收入，提高低收入职工特别是基层一线职工工资水平。大力发展教育事业。促进义务教育均衡发展，加强学前教育和民办教育，整合提升职业教育资源，充分发挥高等教育服务发展作用。市区新建、改扩建30所中小学校，全市新建、改扩建86所公办幼儿园。统筹推进城乡社会保障体系建设。加快推进养老、医疗、失业、工伤、生育保险等“五险合一”进程，进一步提高城乡低保、农村“五保”、医疗救助等补助标准，做到应保尽保。筹建人力资源社会保障综合服务中心。扩大福利服务保障范围，加快市综合性养老服务中心、县（市）级社会福利服务中心、农村敬老院等社会福利设施建设与管理。加强社会救助体系建设，提高对弱势群体的救助服务保障能力。继续加强保障性住房建设。实施经济适用房、公租房、廉租房“三房合一”，全年开工建设各类保障房4.5万套。努力提高人民健康水平。实施“健康郑州人”计划，完善基层医疗卫生服务体系，扩大优质医疗服务供给，打造区域性特色医疗专科，全面建立国家基本药物制度，加快区域性医疗中心建设。持续稳定低生育水平，推进人的全面发展。

　　各位代表！

　　改善民生是各级政府最大的政治。我们将从大处着眼、小处入手，尽最大的努力，更多地减轻所有年轻人和外来务工人员在郑州的创业成本、就业压力、安居负担，更多地改善所有老年人和困难职工群众的生活待遇、医疗待遇、社保待遇，更多地解决所有市民的饮食起居安全、出行休闲方便、消费物美价廉等问题，努力让省会市民生活得更有尊严、更加幸福！

　　（九）持续完善网格化管理，为经济社会持续健康快速发展提供机制保障。继续探索完善以网格为载体坚持依靠群众推进工作落实长效机制，强化“发现问题、反映问题、解决问题、绩效评价、责任查究”机制，着力解决条块深度融合、履职能力提升、依法行政与群众自治融合衔接等问题，不断扩大网格化管理成效。进一步加强和创新社会管理，完善社情民意调查分析机制，畅通群众诉求渠道。健全源头预防的矛盾排查化解体系，落实社会稳定风险评估机制，完善“大调解”工作机制。加大社会管理综合治理力度，加强社会管理治安防控体系建设，推进平安郑州建设。大力推进城乡社区分类管理、达标升级、示范创建，提升社区服务能力。强化公共安全体系和企业安全生产基础建设，大力开展煤矿、非煤矿山、危险化学品、消防、道路交通、建筑工地等重点领域安全整治，坚决遏制重特大事故。强化食品药品监管。切实保障校园校车安全。深入开展“六五”普法，营造知法、用法、守法的良好氛围。

　　继续加强国防教育和国防后备力量建设，支持驻郑部队建设，深入开展双拥共建活动，建立军地军民融合式发展新机制。支持工会、共青团、妇联等人民团体发挥桥梁纽带作用，注重发挥工商联等社团作用。继续做好统计、审计、人防、外事、侨务、对台、民族、宗教、史志、气象、地震、档案、社科和支援新疆哈密等工作。

　　四、加强政府自身建设

　　加强政府自身建设、提高能力、提升水平是推进我市持续健康快速发展的关键所在。我们将认真落实中央改进工作作风、密切联系群众“八项规定”和省委、市委改进工作作风的具体措施，积极开展以“为民、务实、清廉”为主要内容的党的群众路线教育实践活动，努力建设人民满意的高效服务型政府。

　　（一）提高行政效能，努力建设“为民政府”。始终把群众普遍关注的事情作为政府工作的重点，把群众反映最强烈的问题作为改进政府工作的着力点。坚持科学决策、民主决策、依法决策，严格依法行政、依法办事。进一步精简下放行政审批事项，加强行政服务中心建设，积极推行网上办公、网上审批和行政监察，大力推进电子监察系统的建设和应用，不断提升政务服务水平，不断提升政府的行政执行力和公信力。

　　（二）转变工作作风，努力建设“务实政府”。坚持问政于民、问需于民、问计于民，办好顺民意、解民忧、惠民生的实事。坚持求真务实，深入实际、深入基层、深入群众调查研究，切实解决实际问题。大力精简会议活动、文件简报、检查评比、迎来送往，真正把精力用到谋发展、抓落实上。深入开展争创“人民满意的公务员集体”、“人民满意的公务员”活动，努力打造一支作风硬、素质高、能力强的公务员队伍。

　　（三）自觉接受监督，努力建设“清廉政府”。严格执行人大及其常委会的决议决定，自觉接受法律和工作监督，主动接受政协民主监督，认真办理人大代表议案、建议和政协委员提案，广泛听取民主党派、无党派人士、人民团体意见，虚心接受社会监督和舆论监督，让人民更有效监督政府，使政府更好地服务人民。严格落实党风廉政建设责任制，全面加强惩治和预防腐败体系建设，深入推进政务公开，让权力在阳光下运行，坚决反对特权思想、特权现象。厉行勤俭节约，坚决制止铺张浪费，坚决反对讲排场比阔气，坚决抵制享乐主义和奢靡之风！

　　各位代表！

　　中原经济区郑州都市区建设在新的起点上迈入关键阶段。面对全市人民的新期盼，我们深感责任重大、使命光荣！让我们紧密地团结在以习近平同志为总书记的党中央周围，在省委、省政府和市委的坚强领导下，凝心聚力，攻坚克难，务实重干，为把郑州早日建设成为自然之美、社会公正、城乡和谐的现代化都市区，率先全面建成小康社会而努力奋斗！
